# Self-expanding open-cell carotid stent for symptomatic moderate-to-severe vertebral artery origin atherosclerotic stenosis: a single-center retrospective study

**DOI:** 10.3389/fneur.2026.1805607

**Published:** 2026-07-14

**Authors:** Hongliang Zhong, Xiaodong Shi, Jianwen Jia, Hongchao Yang, Tong Li, Yang Wang, Yongquan Sun, He Liu

**Affiliations:** 1 Department of Neurosurgery, Beijing Chaoyang Hospital, Capital Medical University, Beijing, China; 2 Department of Neurology, Weixian People’s Hospital, Handan, Hebei, China

**Keywords:** atherosclerosis, in-stent restenosis, open-cell design, self-expanding stent, vertebral artery origin stenosis

## Abstract

**Background:**

Open-cell self-expanding stents have been used for vertebral artery origin (VAO) atherosclerotic stenosis; however, data on their safety and durability remain limited.

**Methods:**

We retrospectively screened 96 patients who were evaluated for potential open-cell self-expanding stent treatment of angiographically confirmed VAO stenosis between February 2015 and December 2020. After exclusion of 62 patients (47 with vertebral artery diameter <4.0 mm and 15 with vertebral artery diameter > = 4.0 mm who declined self-expanding stent treatment), the final treated cohort comprised 34 consecutive eligible symptomatic patients who underwent open-cell self-expanding stent implantation. Clinical outcomes were assessed perioperatively and during follow-up. The 30-day safety endpoint included stroke, myocardial infarction, and death. ISR was evaluated among patients who completed imaging follow-up; DSA-confirmed ISR and ISR among all patients with imaging surveillance were both reported.

**Results:**

Of 96 screened patients, 34 symptomatic treated patients were included. Three patients entered the symptomatic 50–69% stenosis pathway after insufficient response to medical therapy, and 31 entered the symptomatic > = 70% high-risk anatomic stenosis pathway. Technical success was 100%. VAO stenosis improved from 75.7 +/− 7.9% preprocedure to 8.4 +/− 7.6% immediately after stenting (*p* < 0.001). Distal embolic protection was used in all procedures. Periprocedural complications occurred in 2/34 patients (5.9%; 95% CI, 1.6–19.1%), including 1 symptomatic acute cerebral infarction. No myocardial infarction or death occurred within 30 days. Clinical follow-up was available in 31/34 patients (91.2%); long-term telephone follow-up had a median duration of 43.0 months (IQR, 35.0–55.0; range, 10–83), with no treatment-requiring recurrent symptoms reported. Imaging follow-up was available in 24/34 patients (70.6%) for a median of 6.0 months (IQR, 3.8–13.5; range, 1–60). DSA-confirmed ISR occurred in 1/16 patients (6.3%; 95% CI, 1.1–28.3%) who underwent DSA follow-up; among all patients with imaging surveillance, ISR was observed in 1/24 (4.2%; 95% CI, 0.7–20.2%).

**Conclusion:**

In this single-center retrospective series, open-cell self-expanding stent implantation for atherosclerotic VAO stenosis appeared technically feasible and was associated with infrequent periprocedural adverse events. Longer-term durability and optimal patient selection require confirmation in larger prospective studies with standardized imaging follow-up.

## Introduction

Posterior circulation ischemic stroke and transient ischemic attack (TIA) account for a substantial proportion of ischemic cerebrovascular events, and atherosclerotic disease of the vertebrobasilar system is an important underlying mechanism (1–4). Among extracranial lesions, atherosclerotic stenosis at the vertebral artery origin (VAO) is frequently implicated in posterior circulation hypoperfusion or artery-to-artery embolism and has been associated with a considerable long-term risk of recurrent ischemic events despite contemporary secondary prevention strategies (1–4).

Best medical therapy and aggressive risk-factor modification remain the foundation of management. However, for selected patients with symptomatic moderate-to-severe VAO stenosis or anatomically high-risk severe stenosis with insufficient collateral compensation, recurrent ischemic symptoms may persist under optimized medical therapy, and revascularization is often considered. Surgical reconstruction for VAO stenosis may carry relatively high morbidity in some series, which has driven interest in endovascular angioplasty and stenting as a less invasive alternative (1).

Endovascular treatment of VAO stenosis has demonstrated high technical success, yet durability remains a key challenge. Balloon-expandable stents are widely used in clinical practice; nevertheless, reported in-stent restenosis (ISR) rates vary widely, and mechanical issues such as deformation or fracture have been described, potentially related to elastic recoil and the dynamic motion at the VAO-subclavian junction during respiration and neck movement (1, 5–13).

In this setting, nitinol self-expanding carotid stents with an open-cell design may offer theoretical advantages, including improved conformability, sustained radial force, and fatigue resistance, which could be beneficial for accommodating vessel motion and diameter transitions at the VAO (8, 14). However, data regarding the safety and longer-term performance of open-cell self-expanding stents specifically for VAO atherosclerotic stenosis remain limited.

Therefore, we conducted a single-center retrospective study to evaluate the feasibility, periprocedural safety, and follow-up outcomes of open-cell self-expanding stent implantation for moderate-to-severe atherosclerotic VAO stenosis in patients with a target vertebral artery diameter > = 4.0 mm. Clinical outcomes and imaging findings were assessed perioperatively and during follow-up, with particular attention to ISR among patients who completed imaging surveillance.

Although this study was conducted in a neurointerventional setting, VAO stenosis is an atherosclerotic vascular disease with substantial relevance to vascular medicine because patient selection, risk-factor burden, antithrombotic management, device choice, and restenosis are shared concerns across cerebrovascular and cardiovascular practice.

## Methods

### Study design and ethics

A single-center retrospective study was conducted at Beijing Chaoyang Hospital, Capital Medical University. Consecutive patients who underwent endovascular stent implantation for atherosclerotic stenosis at the vertebral artery origin (VAO) between February 2015 and December 2020 were reviewed.

The study protocol was approved by the Ethics Committee of Beijing Chaoyang Hospital, Capital Medical University. Written informed consent was obtained from patients and/or their family members.

### Study population and inclusion criteria

Patients were eligible if atherosclerotic VAO stenosis was confirmed by digital subtraction angiography (DSA), if the target vertebral artery diameter was > = 4.0 mm, and if posterior-circulation ischemic symptoms judged clinically attributable to the target VAO lesion or symptomatic high-risk anatomy supported revascularization after multidisciplinary assessment. The diameter threshold was used because the open-cell carotid stent platform and the vertebral-subclavian transition require adequate target-vessel caliber for stent apposition and to reduce the risk of edge mismatch; this criterion also means that the findings should not be generalized to patients with smaller vertebral arteries. VAO tortuosity was also considered during patient and device selection.

Two enrollment pathways were applied, and all included patients had posterior-circulation symptoms before intervention. First, symptomatic moderate stenosis was defined as VAO stenosis > = 50% with clearly related clinical symptoms (recurrent transient ischemic attacks or ischemic stroke) and poor response after > = 3 months of standardized medical therapy. Poor response was defined as recurrent or persistent posterior-circulation ischemic symptoms despite antiplatelet therapy, statin treatment, and risk-factor management. Second, symptomatic high-risk anatomic severe stenosis was defined as VAO stenosis > = 70% with posterior-circulation symptoms and a high estimated risk of recurrent ischemia because the contralateral vertebral artery was non-dominant, did not contribute to basilar artery perfusion, or was occluded. This risk profile was determined after assessment by at least two specialist physicians. Standardized medical management, including antiplatelet therapy, statin therapy, and risk-factor control, was used before intervention when clinically feasible; a fixed > = 3-month medical-therapy failure period was required for the symptomatic 50–69% pathway but was not mandatory for the > = 70% high-risk pathway. In the final cohort, 3 patients were included through the symptomatic 50–69% pathway and 31 through the symptomatic > = 70% high-risk pathway.

Exclusion criteria were as follows: (1) non-atherosclerotic stenosis; (2) vertebral artery diameter <4.0 mm; (3) acute infarction related to the target vertebral artery stenosis within 3 weeks before the procedure; and (4) contraindications to vertebral artery angioplasty/stenting (e.g., intracranial hemorrhage, severe contrast allergy, allergy to antiplatelet agents, severe comorbid cardiopulmonary or other systemic disease, or an expected survival <2 years).

### Imaging assessment and lesion definition

Preprocedural evaluation included head computed tomography and/or magnetic resonance imaging to assess intracranial infarction, vascular calcification/plaque, and stenosis. Diagnostic DSA was performed in all patients. Aortic arch angiography and bilateral carotid and subclavian artery angiography were obtained to assess stenosis severity and collateral circulation.

The degree of VAO stenosis was measured according to the North American Symptomatic Carotid Endarterectomy Trial (NASCET) method: (reference diameter distal to the stenosis—minimal luminal diameter at the stenosis)/reference diameter distal to the stenosis (15).

### Endovascular procedure

Continuous electrocardiographic monitoring was used intraoperatively. Local anesthesia was administered. Femoral artery access was obtained using a modified Seldinger technique, and an 8-F arterial sheath was inserted. Systemic heparinization was performed.

An 8-F guiding catheter (MPA1; Cordis, United States) was advanced to the origin of the ipsilateral subclavian artery over a 0.035-inch guidewire. In patients with insufficient guiding-catheter stability, an additional microguidewire was advanced into the brachial artery through the guiding catheter to improve support.

A distal embolic protection device (Spider FX; Medtronic, United States) was deployed in the extracranial segment of the ipsilateral vertebral artery at approximately the C2 vertebral body level in all cases. Balloon angioplasty was performed at the stenotic segment before stent deployment, with balloon size selected according to lesion morphology, target-vessel diameter, and whether staged dilation was required for near-occlusion or tandem disease. After satisfactory lesion preparation, an open-cell self-expanding carotid stent (Protege; Medtronic, United States) was deployed with the distal end positioned beyond the stenosis and the proximal end extending to the mid segment of the subclavian artery to fully cover the lesion. A tapered stent was selected when there was an obvious diameter difference between the vertebral artery and the subclavian artery; otherwise, a straight stent was selected.

Post-deployment angiography was performed to assess stent apposition, distal embolization, vasospasm, flow delay, dissection, and residual stenosis. Balloon post-dilation of the stented segment was performed selectively when stent apposition was unsatisfactory or when residual stenosis was > = 20% until adequate patency was confirmed angiographically.

### Antiplatelet regimen and platelet function testing

Aspirin (100 mg/day), clopidogrel (75 mg/day), and atorvastatin (20 mg/day) were administered for 3–5 days before the procedure. Genetic testing for aspirin and clopidogrel metabolism was completed, and platelet function inhibition was assessed using thromboelastography. Target inhibition thresholds were an arachidonic acid (AA) inhibition rate > = 50% and an adenosine diphosphate (ADP) inhibition rate > = 30%.

If the ADP inhibition target was not achieved, the clopidogrel dose was doubled (150 mg/day) before and after the procedure or clopidogrel was replaced with cilostazol (50 mg twice daily).

Intraoperative systemic heparinization was routinely used. After the procedure, low-molecular-weight heparin (3,800 U every 12 h) was administered for 3 days. Aspirin (100 mg/day) plus clopidogrel (75 mg/day) was continued for 6 months, after which aspirin monotherapy was maintained long term.

### Outcome definitions

Periprocedural imaging surveillance was performed using routine postprocedural head CT and brain MRI to determine whether intracranial hemorrhage or acute cerebral infarction had occurred.

The primary 30-day safety endpoint was defined as any stroke, myocardial infarction, or death within 30 days after the procedure. Stroke was recorded as symptomatic ischemic or hemorrhagic stroke; silent punctate lesions detected on routine MRI were documented separately and were not counted as symptomatic stroke unless accompanied by corresponding clinical symptoms.

TIA, recurrent posterior circulation ischemic symptoms, access-site complications, intracranial hemorrhage, device-related complications, reintervention, and mortality during follow-up were also recorded when available.

### Follow-up and missing data handling

Clinical follow-up was scheduled at 2 weeks, 3 months, and 6 months after the procedure. Antiplatelet use, symptoms, neurological examination findings, and mortality were documented.

Imaging follow-up was scheduled as follows: DSA at 3–6 months; and cervical vascular ultrasound or computed tomography angiography annually from 1 year onward. In routine follow-up, ISR was primarily determined by DSA. For patients followed by CTA/MRA or ultrasound, suspected restenosis or stent morphology change prompted confirmatory DSA whenever clinically feasible.

In-stent restenosis (ISR) was defined as > = 50% luminal stenosis on follow-up DSA. Noninvasive CTA/MRA or ultrasound results were used for surveillance and were interpreted cautiously when DSA confirmation was unavailable.

Missing follow-up data were handled descriptively. Clinical and imaging outcomes were summarized using the available denominators at each time point, and ISR was assessed only among patients who completed imaging follow-up; no data imputation was performed.

### Statistical analysis

Statistical analyses were performed using SPSS (version 23.0). Continuous variables with an approximately normal distribution were presented as mean +/− standard deviation.

For paired comparisons of stenosis severity before and immediately after stent implantation, a paired-sample *t* test was used, consistent with the original analysis plan. A two-sided *p* value <0.05 was considered statistically significant.

Where applicable, 95% confidence intervals for proportions were calculated using the Wilson method and reported descriptively. Baseline characteristics were compared between patients with and without imaging follow-up using *t* tests for continuous variables and chi-square or Fisher exact tests for categorical variables as appropriate. Because of the small sample size and very low number of outcome events, no multivariable or factor-association model was performed.

## Results

### Baseline characteristics

**Figure 1:**
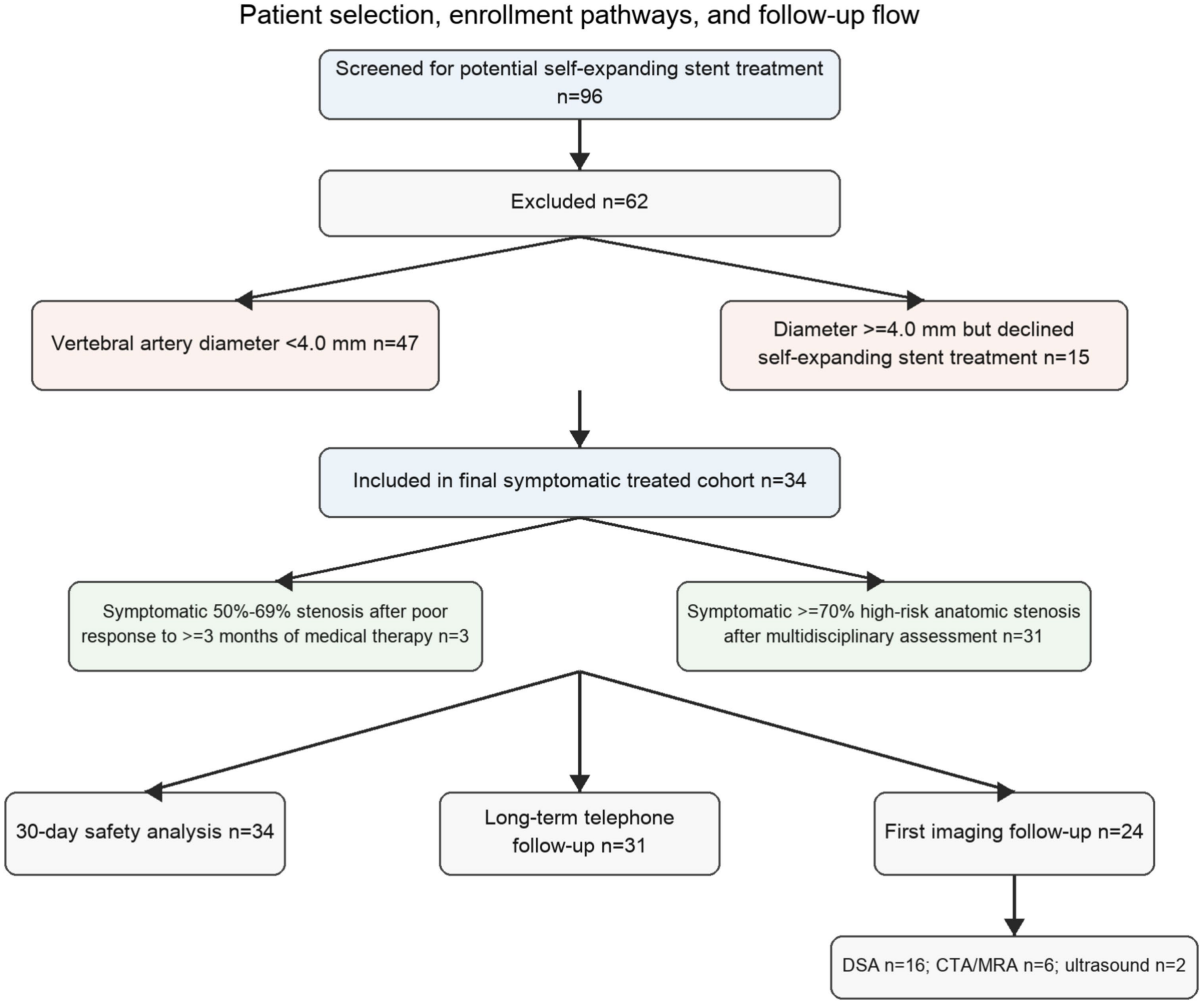
Patient selection, enrollment pathways, and follow-up flow.

A total of 96 patients were screened from the potential self-expanding stent treatment population according to the preliminary treatment criteria. Sixty-two patients were excluded: 47 because the vertebral artery diameter was <4.0 mm and 15 because the vertebral artery diameter was > = 4.0 mm but the patient or family declined self-expanding stent treatment. The final treated cohort included 34 consecutive eligible symptomatic patients [31 men (91.2%) and 3 women (8.8%)], with an age range of 41–84 years (mean 63.85 +/− 9.06 years). The patient-selection, enrollment-pathway, and follow-up flow is shown in Figure 1 to clarify available denominators for safety, clinical follow-up, and imaging follow-up. Baseline demographics and clinical characteristics are summarized in Table 1.

**Table 1:** Baseline characteristics of the overall cohort and comparison according to imaging follow-up status.

Variable	Overall cohort (*N* = 34)	With imaging follow-up (*n* = 24)	Without imaging follow-up (*n* = 10)	*P* value
Age, years	63.85 +/− 9.06 (41–84)	64.8 +/− 8.2	61.6 +/− 11.1	0.425
Male sex	31 (91.2%)	22 (91.7%)	9 (90.0%)	1.000
Stenosis rate, %	75.7 +/− 7.9	75.8 +/− 8.5	75.4 +/− 6.7	0.876
Lesion length, mm	7.4 +/− 6.0	7.7 +/− 6.7	6.7 +/− 4.1	0.590
Vertebral artery diameter, mm	4.8 +/− 0.7	4.9 +/− 0.7	4.7 +/− 0.6	0.339
Hypertension	25 (73.5%)	17 (70.8%)	8 (80.0%)	0.692
Type 2 diabetes	16 (47.1%)	9 (37.5%)	7 (70.0%)	0.134
Dyslipidemia	7 (20.6%)	5 (20.8%)	2 (20.0%)	1.000
Hyperhomocysteinemia	15 (44.1%)	10 (41.7%)	5 (50.0%)	0.728
Coronary heart disease	9 (26.5%)	6 (25.0%)	3 (30.0%)	1.000
Prior cerebral infarction	26 (76.5%)	18 (75.0%)	8 (80.0%)	1.000
Carotid-system stenosis or occlusion	13 (38.2%)	9 (37.5%)	4 (40.0%)	1.000
Left-sided lesion	16 (47.1%)	11 (45.8%)	5 (50.0%)	1.000
Right-sided lesion	18 (52.9%)	13 (54.2%)	5 (50.0%)	1.000

Values are *n* (%) or mean +/− SD unless otherwise indicated. *p* values compare patients with and without imaging follow-up.

At baseline, 16 patients (47.0%) were overweight (body mass index >24). Vascular risk factors included hypertension in 25 (73.5%), type 2 diabetes in 16 (47.1%), dyslipidemia/hyperlipidemia in 7 (20.6%), hyperhomocysteinemia in 15 (44.1%), and coronary heart disease in 9 (26.5%). A smoking history was documented in 26 patients (76.5%), with a duration of 20–60 years (mean 39.2 +/− 11.4 years) and a consumption of 10–40 cigarettes/day (mean 22.5 +/− 7.8 cigarettes/day). Prior cerebral infarction was reported in 26 patients (76.5%), and carotid-system stenosis or occlusion was present in 13 patients (38.2%).

Presenting manifestations included transient ischemic attack in 18 patients (52.9%), posterior circulation cerebral infarction in 14 (41.2%), and dizziness in 2 (5.9%). The target lesion was located on the left side in 16 patients (47.1%) and on the right side in 18 (52.9%); 21 patients (61.7%) had stenosis on the dominant vertebral artery side. VAO tortuosity was present in 12 patients (35.3%) and was considered during treatment planning. Contralateral vertebral artery abnormalities were present in 22 patients (64.7%), including hypoplasia or occlusion in 7, moderate-to-severe stenosis in 11, and continuation as the posterior inferior cerebellar artery without contribution to basilar artery perfusion in 4. Tandem stenosis was observed in 4 patients, including vertebral artery origin plus distal tandem stenosis in 1 and subclavian artery plus vertebral artery origin tandem stenosis in 3. Baseline comparison between patients with and without imaging follow-up did not show statistically significant differences in the available variables (Table 1), although the small subgroup sizes limit inference.

CYP2C19 genotyping for clopidogrel metabolism was completed in 31 patients, with 4 identified as poor metabolizers. Thromboelastography was performed in 30 patients; arachidonic acid inhibition did not reach the target threshold in 2 patients, and adenosine diphosphate inhibition did not reach the target threshold in 2 patients.

**Figure 2:**
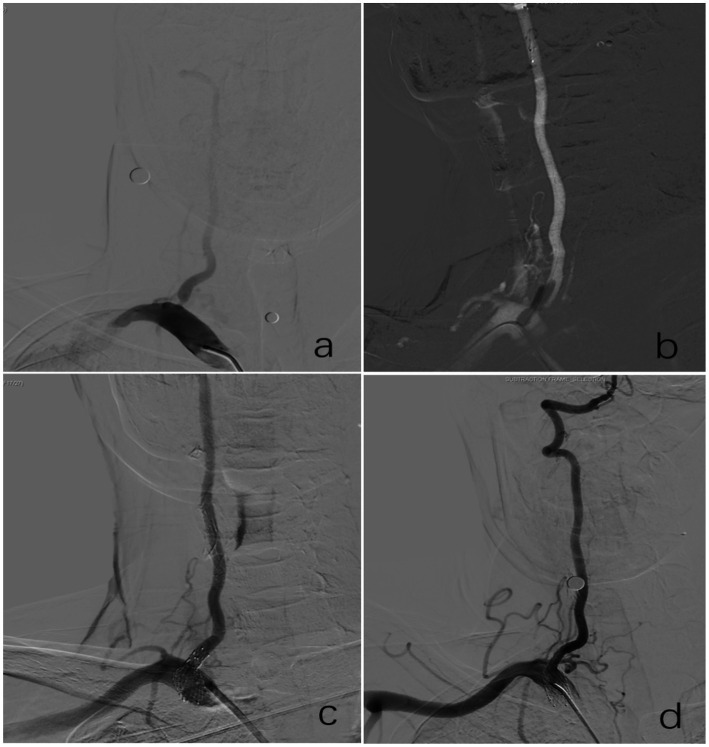
Representative case of severe right vertebral artery origin stenosis treated with a Protege 9 × 30-mm stent. **(a)** Preprocedural angiography shows severe stenosis at the right vertebral artery origin. **(b)** After stent deployment, angiography shows improved lumen caliber. **(c)** Immediate postprocedural angiography confirms adequate patency and lesion coverage. **(d)** Six-month follow-up angiography shows maintained patency without significant restenosis.

A representative case is shown in Figure 2.

### Procedural details

Preprocedural vertebral artery origin stenosis severity ranged from 60 to 100%, with a mean of 75.7 +/− 7.9%. Lesion length ranged from 2.0 to 34.8 mm (mean 7.4 +/− 6.0 mm). The target vertebral artery diameter ranged from 4.0 to 6.8 mm (mean 4.8 +/− 0.7 mm), and the subclavian artery diameter ranged from 6.3 to 13.5 mm (mean 9.9 +/− 1.8 mm).

Technical success was 100% (34/34; 95% CI, 89.8–100%). Distal embolic protection was used in all procedures. A straight stent was used in 20 patients and a tapered stent in 14 patients. Immediately after stent deployment, residual stenosis ranged from 0 to 24% (mean 8.4 +/− 7.6%); the paired comparison with preprocedural stenosis was *p* < 0.001. Thirty-two patients (94.1%) received 1 stent, and 2 patients received 2 stents. Balloon and stent size distributions are summarized in Table 2. Multiple-balloon strategies were used in 4 patients, including VAO plus intracranial tandem disease treated with a 2.5 × 15-mm and 4 × 20-mm balloon strategy; tandem subclavian artery and VAO stenoses treated with 6 × 20-mm and 4 × 20-mm balloons, with the 4-mm balloon used for VAO dilation and the 6-mm balloon used for subclavian stenosis; vertebral artery occlusion requiring stepwise dilation with 1.5 × 15-mm, 2.5 × 15-mm, and 4 × 20-mm balloons; and near-occlusion requiring stepwise dilation with 2.0 × 15-mm and 4 × 20-mm balloons. The two-stent cases included 1 patient treated with an intracranial vertebral artery stent plus a VAO stent and 1 patient who required an additional V2-segment stent for intraoperative dissection. Available procedural records did not identify routine access-site bleeding, clinically significant vasospasm, bradycardia, hypotension, or distal embolization beyond the complications described below.

**Table 2:** Balloon and stent size distributions.

Device category	Size/model	Number	Percentage
Balloon	5 × 20 mm	18	52.9%
Balloon	4 × 20 mm	8	23.5%
Balloon	Multiple-balloon strategy	4	11.8%
Balloon	6 × 20 mm	2	5.9%
Balloon	4 × 30 mm	1	2.9%
Balloon	5 × 30 mm	1	2.9%
Stent	10–7 × 40 mm	9	26.5%
Stent	9 × 30 mm	7	20.6%
Stent	8–6 × 40 mm	5	14.7%
Stent	9 × 40 mm	5	14.7%
Stent	8 × 40 mm	3	8.8%
Stent	8 × 30 mm	2	5.9%
Stent	7 × 40 mm	1	2.9%
Stent	Multiple-stent strategy	2	5.9%

### Primary safety outcomes within 30 days

Within 30 days after the procedure, 1 patient developed symptomatic acute cerebral infarction, presenting with memory decline; memory decline was alleviated after infusion therapy. The 30-day symptomatic stroke rate was 1/34 (2.9%; 95% CI, 0.5–14.9%). No myocardial infarction or death occurred within 30 days, so the composite 30-day safety endpoint occurred in 1/34 patients (2.9%; 95% CI, 0.5–14.9%). No intracranial hemorrhage was documented. No additional silent infarcts were separately documented in the available postoperative MRI records.

No 30-day TIA or posterior circulation ischemic recurrence was documented apart from the symptomatic acute cerebral infarction described above.

### Periprocedural complications

Periprocedural complications occurred in 2 of 34 patients (5.9%; 95% CI, 1.6–19.1%). One patient developed a V2-segment dissection during deployment of the distal embolic protection device and underwent stent placement; vertebral artery origin stenting was subsequently completed. One patient developed symptomatic acute cerebral infarction with memory decline, as described above. No periprocedural death, myocardial infarction, or intracranial hemorrhage was recorded.

### Follow-up completeness

Clinical follow-up was obtained in 31 patients (91.2%) via outpatient visits or telephone contact; clinical follow-up data were unavailable for 3 patients. Among patients with clinical follow-up, symptom status after the procedure was documented as alleviated or resolved in 30 patients, and short-term memory impairment was reported as alleviated in 1 patient who had postoperative temporal lobe infarction. A second long-term telephone follow-up was available in 31 patients, with a median duration of 43.0 months (IQR, 35.0–55.0; range, 10–83). During this long-term telephone follow-up, no treatment-requiring recurrent symptoms were reported. No long-term death, recurrent stroke/TIA, or repeat target-lesion revascularization other than the ISR angioplasty described below was documented in the available follow-up records.

Imaging follow-up was available in 24 patients (70.6%); imaging follow-up data were unavailable for 10 patients. The first imaging follow-up duration had a median of 6.0 months (IQR, 3.8–13.5; range, 1–60). Among those with imaging follow-up (*n* = 24), modalities included DSA in 16 patients (66.7%), CTA/MRA in 6 patients, and cervical vascular ultrasound in 2 patients. Follow-up duration, duration strata, and modality distribution are summarized in Table 3. The reason for absent imaging follow-up was not consistently recorded in the retrospective dataset.

**Table 3:** Follow-up duration, strata, and modality distribution.

Follow-up type	Available patients	Median (IQR), months	Range, months	Follow-up strata	Imaging modality
First imaging follow-up	24/34 (70.6%)	6.0 (3.8–13.5)	1–60	<3 mo: 3; 3–6 mo: 11; 7–12 mo: 4; 13–24 mo: 2; >24 mo: 4	DSA: 16; CTA/MRA: 6; ultrasound: 2
Long-term telephone follow-up	31/34 (91.2%)	43.0 (35.0–55.0)	10–83	<12 mo: 1; 12–24 mo: 1; 25–36 mo: 7; 37–60 mo: 18; >60 mo: 4	Not applicable

IQR, interquartile range; DSA, digital subtraction angiography; CTA, computed tomography angiography; MRA, magnetic resonance angiography. Imaging modality distribution is reported overall because modality-by-time-stratum cross-tabulation was not consistently reconstructable from the retrospective records.

### In-stent restenosis and reintervention

DSA-confirmed in-stent restenosis occurred in 1 of 16 patients who underwent DSA follow-up (6.3%; 95% CI, 1.1–28.3%). This event was identified by DSA at 6 months after the procedure, and balloon angioplasty was performed. Among all 24 patients with imaging surveillance, ISR was observed in 1 patient (4.2%; 95% CI, 0.7–20.2%). Patients followed only by CTA/MRA or ultrasound were not counted as having DSA-confirmed ISR; these noninvasive examinations were used for surveillance and interpreted cautiously when DSA confirmation was unavailable. Because ISR was observed in only 1 patient and imaging follow-up was incomplete and heterogeneous, these estimates should be interpreted as descriptive and hypothesis-generating.

Stent migration was observed in 2 of 24 patients with imaging follow-up (8.3%; 95% CI, 2.3–25.9%). In both cases, migration was detected by DSA at 1 month after the procedure. Compared with immediate postprocedural angiography, the stent had shifted approximately 2 mm toward the subclavian artery, but the stenotic VAO segment remained covered and no restenosis was observed. Both patients were managed with continued observation and had no related symptoms during long-term telephone follow-up at 37 and 57 months. Available records did not allow a reliable analysis of migration mechanism by straight versus tapered stent, lesion length, or vessel-diameter mismatch.

Procedural, device, and follow-up outcomes are summarized in Tables 2–4.

**Table 4:** Procedural, angiographic, and clinical outcomes.

Parameter	Value	95% CI or note
Technical success	34/34 (100%)	89.8–100%
Distal embolic protection used	34/34 (100%)	
Preprocedural stenosis, %	75.7 +/− 7.9	Range, 60–100
Residual stenosis after stenting, %	8.4 +/− 7.6	Range, 0–24
Subclavian artery diameter, mm	9.9 +/− 1.8	Range, 6.3–13.5
Periprocedural complications	2/34 (5.9%)	1.6–19.1%
30-day symptomatic stroke	1/34 (2.9%)	0.5–14.9%
30-day myocardial infarction	0/34 (0%)	0–10.2%
30-day death	0/34 (0%)	0–10.2%
DSA-confirmed ISR	1/16 (6.3%)	1.1–28.3%
ISR among all patients with imaging surveillance	1/24 (4.2%)	0.7–20.2%
Stent migration among patients with imaging follow-up	2/24 (8.3%)	2.3–25.9%

CI, confidence interval; ISR, in-stent restenosis. Confidence intervals for proportions were calculated using the Wilson method.

## Discussion

### Principal findings

In this single-center retrospective cohort of symptomatic patients with moderate-to-severe atherosclerotic stenosis at the vertebral artery origin (VAO) and a target vertebral artery diameter > = 4.0 mm, deployment of an open-cell self-expanding carotid stent at the VAO was technically feasible with a high procedural success rate. Marked angiographic luminal gain was achieved immediately after treatment, while periprocedural adverse events were infrequent. During follow-up (available in 91.2% clinically and 70.6% by imaging), DSA-confirmed ISR was observed in 1 of 16 patients with DSA follow-up (6.3%), ISR among all patients with imaging surveillance was 1 of 24 (4.2%), and stent migration was observed in 2 of 24 (8.3%). Long-term telephone follow-up in 31 patients reached a median of 43.0 months (IQR, 35.0–55.0) and did not identify treatment-requiring recurrent symptoms. These findings should be interpreted as descriptive feasibility and safety observations rather than evidence of comparative effectiveness.

### Mechanistic considerations

VAO lesions are commonly located at a hemodynamically complex segment, which may be related to relatively low flow and local turbulence at the origin (16). When symptoms recur despite medical therapy, revascularization is often considered, whereas open surgical reconstruction has been associated with relatively high complication and mortality rates (1). Endovascular angioplasty/stenting has therefore been used as an alternative strategy since early reports in the 1990s (17).

Nevertheless, ISR remains a key concern, and prior work suggests that elastic recoil and continuous motion of the vertebral artery and subclavian artery may contribute to restenosis at the ostium (11). From a device-mechanics perspective, an open-cell nitinol self-expanding stent may provide sustained radial force and conformability, which could potentially improve apposition in a dynamic segment and might reduce mechanical fatigue-related deformation over time (8, 14).

In contrast, balloon-expandable stents have been reported to experience metal fatigue and may provide less tolerance to repetitive respiratory and neck-motion stresses, which could potentially be linked with deformation or fracture and subsequent restenosis in some settings (9–13).

In addition, the deployment strategy used here (extending from the vertebral artery into the subclavian artery) might potentially improve coverage of plaque at the VAO-subclavian junction and could reduce edge-related recoil, although these interpretations remain hypothesis-generating.

### Comparison with previous studies

Published series of VAO stenting have consistently demonstrated high technical success rates; however, reported ISR rates following balloon-expandable or coronary-type stents have varied substantially across studies, likely reflecting differences in patient selection, lesion characteristics, antiplatelet regimens, stent platforms, and follow-up protocols (5, 6, 13, 18–20). In the present study, the observed ISR rate among patients with imaging follow-up (1/24, 4.2%) was numerically low. Nevertheless, such cross-study comparisons should be interpreted with caution given the lack of a contemporaneous comparator group, the considerable heterogeneity in lesion morphology and antiplatelet strategies, and the incompleteness and modality heterogeneity of follow-up across published series.

Previous investigations have further suggested that anatomical factors, including vertebral artery diameter and vascular tortuosity, may significantly influence ISR risk at the VAO (6, 7, 12). Because all patients in the current cohort had a target vessel diameter > = 4.0 mm, the relatively low ISR frequency observed may partly reflect patient selection rather than a definitive device-specific effect.

With respect to self-expanding stents, the ISR and periprocedural complication rates observed in this study fall within the range reported by other series evaluating self-expanding platforms for VAO lesions (14, 16, 21, 22). Importantly, available comparative evidence does not uniformly favor self-expanding stents. For instance, Langwieser et al. reported differing conclusions regarding the relative performance of stent types in extracranial vertebral artery disease, underscoring the ongoing uncertainty and the need for cautious interpretation of device-specific outcomes (23).

### Stent migration

Stent migration was observed in 2 patients with imaging follow-up. Both events were detected by DSA at 1 month and consisted of approximately 2-mm displacement toward the subclavian artery compared with immediate postprocedural angiography. In both cases, the stent continued to cover the stenotic segment, no restenosis or clinical consequence was observed, and no additional intervention was required. Long-term telephone follow-up at 37 and 57 months identified no related symptoms. Migration is clinically relevant at the VAO because this segment is exposed to motion, vessel-diameter transition, and mechanical interaction between the vertebral and subclavian arteries. The current dataset does not allow reliable identification of migration predictors, but possible contributors include vessel-diameter mismatch, lesion length, stent configuration, and local motion at the VAO-subclavian junction. These observations support careful stent sizing, full lesion coverage, and structured imaging surveillance in future studies.

### Limitations and clinical implications

Several limitations should be emphasized. First, this was a single-center study with a retrospective design. Second, the sample size was small. Third, there was no contemporaneous control group (e.g., balloon-expandable stenting or best medical therapy), limiting comparative inference. Fourth, follow-up was incomplete, particularly for imaging (available in 70.6%), and ISR was therefore assessable only in patients who underwent imaging follow-up. Fifth, imaging follow-up modalities were heterogeneous; although DSA was the defining standard for ISR, not all followed patients underwent DSA. Therefore, DSA-confirmed ISR and ISR among all patients with imaging surveillance were reported separately, and non-DSA surveillance should not be interpreted as equivalent to negative DSA. Sixth, although baseline characteristics were compared between patients with and without imaging follow-up, the subgroup sizes were small and residual selection bias cannot be excluded. Seventh, the number of ISR, complication, and migration events was too small for reliable multivariable modeling. Eighth, modality-by-time-stratum cross-tabulation and detailed reasons for missing imaging follow-up were not consistently reconstructable from the retrospective records.

These constraints preclude causal interpretation and do not support claims of superiority or broad expansion of indications. Clinically, the current findings suggest that open-cell self-expanding carotid stenting at the VAO may be technically feasible in selected patients with symptomatic or high-risk VAO stenosis and an adequately sized vertebral artery (> = 4.0 mm), when performed in experienced centers with careful periprocedural management. The 4.0-mm diameter threshold may have enriched the cohort for anatomically favorable lesions, and the results should not be extrapolated to smaller vertebral arteries without further evidence. Prospective, controlled studies with standardized imaging follow-up are needed to better define long-term durability and patient selection criteria.

## Conclusion

In selected patients with moderate-to-severe atherosclerotic stenosis at the vertebral artery origin (VAO) and an adequately sized target vertebral artery (> = 4.0 mm), open-cell self-expanding carotid stent deployment appears technically feasible. In this retrospective single-center cohort, periprocedural adverse events were infrequent, suggesting an acceptable short-term safety profile when performed with careful periprocedural management. Nevertheless, given the limited sample size, lack of a control group, and incomplete imaging follow-up, these findings should be considered hypothesis-generating. Larger prospective studies with standardized long-term follow-up are needed to confirm durability and refine patient selection.
